# Preparation and Characterization of Defective TiO_2_. The Effect of the Reaction Environment on Titanium Vacancies Formation

**DOI:** 10.3390/ma13122763

**Published:** 2020-06-18

**Authors:** Zuzanna Bielan, Szymon Dudziak, Agnieszka Sulowska, Daniel Pelczarski, Jacek Ryl, Anna Zielińska-Jurek

**Affiliations:** 1 Department of Process Engineering and Chemical Technology, Faculty of Chemistry, Gdansk University of Technology (GUT), G. Narutowicza 11/12, 80-233 Gdansk, Poland; dudziakszy@gmail.com (S.D.); sulowska.as@gmail.com (A.S.); 2 Department of Physics of Electronic Phenomena, Faculty of Applied Physics and Mathematics, Gdansk University of Technology (GUT), G. Narutowicza 11/12, 80-233 Gdansk, Poland; daniel.pelczarski@pg.edu.pl; 3 Department of Electrochemistry, Corrosion and Materials Engineering, Faculty of Chemistry, Gdansk University of Technology (GUT), G. Narutowicza 11/12, 80-233 Gdansk, Poland; jacryl@pg.edu.pl

**Keywords:** titanium vacancies, HIO_3_, phenol degradation, scavengers, photocatalysis

## Abstract

Among various methods of improving visible light activity of titanium(IV) oxide, the formation of defects and vacancies (both oxygen and titanium) in the crystal structure of TiO_2_ is an easy and relatively cheap alternative to improve the photocatalytic activity. In the presented work, visible light active defective TiO_2_ was obtained by the hydrothermal reaction in the presence of three different oxidizing agents: HIO_3_, H_2_O_2_, and HNO_3_. Further study on the effect of used oxidant and calcination temperature on the physicochemical and photocatalytic properties of defective TiO_2_ was performed. Obtained nanostructures were characterized by X-ray diffractometry (XRD), specific surface area (BET) measurements, UV-Vis diffuse reflectance spectroscopy (DR-UV/Vis), photoluminescence spectroscopy (PL), X-ray photoelectron spectroscopy (XPS), and electron paramagnetic resonance (EPR) spectroscopy. Degradation of phenol as a model pollutant was measured in the range of UV-Vis and Vis irradiation, demonstrating a significant increase of photocatalytic activity of defective TiO_2_ samples above 420 nm, comparing to non-defected TiO_2_. Correlation of EPR, UV-Vis, PL, and photodegradation results revealed that the optimum concentration of HIO_3_ to achieve high photocatalytic activity was in the range of 20–50 mol%. Above that dosage, titanium vacancies amount is too high, and the obtained materials’ photoactivity was significantly decreased. Studies on the photocatalytic mechanism using defective TiO_2_ have also shown that ^•^O_2_^−^ radical is mainly responsible for pollutant degradation.

## 1. Introduction

One of the main challenges of the 21st century is the pollution of the water environment. Compounds such as pharmaceuticals, hormones, or personal care products are detected in surface waters, which negatively affect human health and entire ecosystems [1]. In this regard, advanced oxidation processes (AOPs) allow for the effective removal of impurities from water. Heterogeneous photocatalysis, as one of the AOPs, has gained considerable attention due to effective removal in the presence of light and semiconductor of xenobiotics not susceptible to biological degradation.

In the photocatalytic process, semiconductor absorbs electromagnetic radiation with an energy greater or equal to energy bandgap. The generated charge carriers take part at the surface in redox reactions with the water, oxygen, and hydroxyl ion molecules leading to the formation of reactive oxygen species, capable of non-selective and sufficient oxidation of pollutants. Titanium(IV) oxide is one of the most frequently used semiconductors for photocatalysis due to its good photocatalytic activity, cost-effectiveness, non-toxicity, and high stability [2]. However, the use of TiO_2_ in heterogeneous photocatalysis is limited due to charge carriers’ recombination and almost no activity in the visible light.

Several different strategies have been proposed to obtain TiO_2_ active in the range of visible light. Advanced modification of semiconductor materials with metals (Ag [3], Au [4], Mo [5], Fe [6], Pt [7], and Pd [8]), as well as doping with non-metals (N [9], C [10], F [11], Cl [12], and S [13]) and dyes sensitization [14,15,16] enable to obtain heterogeneous photocatalysts active in the visible light. Nonetheless, all the presented methods have several drawbacks. Non-metal-doped semiconductors usually are unstable in long-term processes because of dopant liberation from surface layers [17,18]. Introducing non-metals into the TiO_2_ lattice could also result in the formation of oxygen vacancies, which could act as additional electron-hole pairs recombination centers [19,20]. In turn, doping with metal ions, as well as a surface modification with its nanoparticles, is more expensive and is not cost-effective in photocatalysis scaling-up [21]. Moreover, such nanomaterials often suffer from thermal and optical instability [22]. As for the matter of dye-sensitized semiconductors, widely used sensitizers such as alizarin red S [23], bipyridine complexes [24,25], phthalocyanine [16,26] absorbed on the surface of TiO_2_ could be desorbed during the photocatalytic process and greatly depress the photoactivity [27,28].

Another possibility of increasing the photoactivity of TiO_2_ is an introduction to its crystal structure intrinsic defects. To the category of this self-structural modification belong titanium/oxygen vacancy self-doping and a surface disorder as well as the formation of Ti-OH bonds on the surface layer [29,30]. Great attention to blue (Ti^3+^ defected) and black TiO_2_ is related mostly to extending the light absorption to the visible region [31,32]. As presented by Lettieri et al. [33], blue TiO_2_ could be obtained from commercially available P25 and anatase powders in simple solvent reflux thermal treatment. It allowed to surface and sub-surface oxygen vacancies formation. Consequently, TiO_2_ bandgap has been narrowed to about 2.3 eV and visible light activity was significantly increased. Among all studied titania defects, the most rarely investigated are titanium vacancies and titanium interstitials despite their excellent quadrupole donor and acceptor properties. Revolutionary work in the field of TiO_2_ vacancies was reported by Wu et al. [34]. The yellow, ultra-small defective TiO_2_ was obtained by a simple sol–gel method within 8 h of UV irradiation, without introducing any external dopants. The formed titanium vacancies and titanium interstitials played a crucial role in visible-light-driven H_2_ production from formaldehyde solution, not only initiating but also promoting photocatalytic activity in visible light. Furthermore, cycling tests indicated the stability of yellow defective TiO_2_, compared with normal TiO_2_ assisted with co-catalysts [34].

Phenol is one of the commonly used model organic compounds in photocatalytic wastewater treatment. Its degradation pathway is intensively studied for a thorough understanding of the photocatalytic reaction with the application of various photocatalysts [35,36,37]. Kang et al. studied degradation under visible light using F-doped TiO_2_ hollow nanocubes with oxygen vacancies [38]. After 60 min of irradiation, about 60% of organic contamination was degraded. A different approach was presented by Colón et al. [39]. The titanium(IV) ox0ide photocatalysts obtained from titanium isopropoxide precursor were treated with different inorganic acids and then calcined in temperature range from 400 to 800 °C. It was reported that such treatment was responsible for the generation of oxygen vacancies on the surface of the photocatalysts. The highest photocatalytic activity was noticed for pre-sulfated TiO_2_ calcined in 600 °C [39]. Nevertheless, in the literature there is lack of information concerning the photocatalytic activity of defective TiO_2_ with titanium vacancies under visible or UV-visible light in reaction of phenol degradation.

Therefore, in this study, we propose a simple method of preparation visible light active TiO_2_ with titanium vacancies obtained by a hydrothermal reaction in a suitable oxidizing environment (HIO_3_, H_2_O_2_, and HNO_3_). The most appropriate amount of used oxidant, as well as the durability of generated titanium vacancies were investigated. The effect of introduced defects on physicochemical and photocatalytic properties was studied. The obtained samples were characterized by X-ray diffractometry (XRD), specific surface area (BET) measurements, UV-Vis diffuse reflectance spectroscopy (DR-UV/Vis), photoluminescence spectroscopy (PL), X-ray photoelectron spectroscopy (XPS), and electron paramagnetic resonance (EPR) spectroscopy. The photodegradation of phenol as a model organic pollutant using the obtained photocatalysts was subsequently investigated in the range of UV-Vis and Vis irradiation. Furthermore, the mechanism of phenol degradation and the role of four oxidative species (h^+^, e^−^, ^•^OH, and ^•^O_2_^−^) in the studied photocatalytic process were investigated.

## 2. Materials and Methods 

Titania organic precursor: titanium(IV) butoxide (99+%) was provided by Alfa Aesar (Haverhill, MA, USA). Iodic acid (99.5%), nitric acid (68%), and hydrogen peroxide (30%) were purchased from Sigma (Poznan, Poland) and were used for TiO_2_ structure modification. Acetonitrile and orthophosphoric acid (85%) for HPLC mobile phase preparation were provided by Merck (Darmstadt, Germany) and VWR (Gdansk, Poland), respectively. Phenol, used as a model organic recalcitrant pollutant in photocatalytic activity measurements, was purchased from VWR. All reagents were used without further purification. 

### 2.1. Preparation of Defective TiO_2_ in the Presence of Different Oxidizing Agents

The preparation of defective TiO_2_ was performed by a hydrothermal method and annealing process. Titanium(IV) butoxide (TBT) was used as a TiO_2_ precursor, and iodic acid (HIO_3_), nitric acid (HNO_3_), or hydrogen peroxide (H_2_O_2_) was used as an oxidizing environment. First, an appropriate amount of HIO_3_, HNO_3_, or H_2_O_2_ (as mentioned in Table 1) was dissolved in 80 cm^3^ of distilled water. After that, 10 cm^3^ of TBT was added dropwise, and the obtained suspension was stirred for 1 h at room temperature. In the next step, the suspension was transferred into a Teflon-lined autoclave for thermal treatment at 110 °C for 24 h. The resultant precipitate was centrifuged, dried at 70 °C, and then calcined at 300 °C. Calcination was carried out in two steps: with a heating rate of 3 °C·min^−1^ to the temperature of 180° for 45 min, then with a heating rate of 2 °C·min^−1^ to the temperature of 300° for 3 h. A series of defective TiO_2_ photocatalysts with different content of used oxidants, calculated as amount relative to TiO_2_, are presented in Table 1. For easier recognition of samples, TBT-HIO_3_, TBT-HNO_3_, and TBT-H_2_O_2_ names are assigned to defective photocatalysts obtained in the assistance of HIO_3_, HNO_3_, and H_2_O_2_ oxidants, respectively.

In order to obtain the defective photocatalysts’ series for their thermal stability test, TBT-HIO_3__20 was synthesized hydrothermally, as was reported in the previous paragraph. Further, the dry product was calcined in five different temperatures: 300 °C, 400 °C, 450 °C, 650 °C, and 1000 °C. Calcination was carried out in two steps: with a heating rate of 3 °C·min^−1^ to the temperature of 180° for 45 min, then with a heating rate of 2 °C·min^−1^ to the set temperature for 3 h.

### 2.2. Characterization of Obtained Defective Photocatalysts

The XRD analyses were performed using the Rigaku Intelligent X-ray diffraction system SmartLab (Rigaku Corporation, Tokyo, Japan) equipped with a sealed tube X-ray generator (a copper target; operated at 40 kV and 30 mA). Data was collected in 2θ range of 5–80° with a scan speed and scan step of 1°·min^−1^ and 0.01°, respectively. The analyses were based on the International Centre for Diffraction Data (ICDD) database. The crystallite size of the photocatalysts in the vertical direction to the corresponding lattice plane was determined using the Scherrer’s equation with the Scherrer’s constant equal to 0.891. Quantitative analysis, including phase composition with standard deviation, was calculated using the reference intensity ratio (RIR) method from the most intensive independent peak of each phase.

Nitrogen adsorption–desorption isotherms (BET method for the specific surface area) were recorded using the Micromeritics Gemini V (model 2365; Norcross, GA, USA) instrument at 77 K (liquid nitrogen temperature).

Light absorption properties were measured using diffuse reflectance (DR) spectroscopy in the range of 200–800 nm. The bandgap energy of obtained samples was calculated from (F(R)·E)^0.5^ against E graph, where E is the photon energy, and F(R) is the Kubelka–Munk function, proportional to the radiation’s absorption. The measurements were carried out using ThermoScientific Evolution 220 Spectrophotometer (Waltham, MA, USA) equipped with a PIN-757 integrating sphere. As a reference, BaSO_4_ was used.

X-ray photoelectron spectroscopy (XPS) measurements were conducted using Escalab 250Xi multi-spectrometer (Thermofisher Scientific, Walthman, MA, USA) using Mg K X-rays. Photoluminescence (PL) spectra were recorded on a Perkin-Elmer LS 55 fluorescence spectrometer (Waltham, MA, USA) employing Xenon discharge lamp equivalent to 20 kW as the excitation source. The samples were excited at 250 nm in the air at room temperature. A 290 nm cut off filter was used during measurements at range 300–700 nm.

Electron paramagnetic resonance (EPR) spectroscopy was used for intrinsic defects formation confirmation. Measurements were conducted using RADIOPAN SE/X-2547 spectrometer (Poznań, Poland), operating at room temperature, with frequency in range 8.910984–8.917817 GHz.

Defective TiO_2_ photocatalysts morphology was determined by scanning electron microscopy (SEM) equipped with energy-dispersive X-ray spectroscopy (EDS; HITACHI, S-3400N, Tokyo, Japan).

### 2.3. Measurements of Photocatalytic Activity

Photocatalytic activity of the obtained samples was evaluated in phenol degradation reaction, both in UV-Vis and Vis light irradiation, using 300 W Xenon lamp (LOT Oriel, Darmstadt, Germany). For the visible light measurements, a cut-off 420 nm filter (Optel, Opole, Poland) was used to obtain a settled irradiation interval. A 0.05 g (2 g·dm^−3^) of a photocatalyst, together with a 20 mg·dm^−3^ phenol solution, was added to a 25 cm^3^ quartz photoreactor with an exposure layer thickness of 3 cm and obtained suspension was stirred in darkness for 30 min to provide adsorption–desorption equilibrium. After that, photocatalyst suspension was irradiated under continuous stirring and a power flux of 30 mW·cm^−2^ for 60 min. The constant temperature of the aqueous phase was kept at 20 °C using a water bath. Every 20 min of irradiation, 1.0 cm^3^ of suspension was collected and filtered through syringe filters (pore size = 0.2 µm) for the removal of photocatalysts particles. Phenol concentration, as well as a formation of degradation intermediates, were analyzed using reversed-phase high-performance liquid chromatography (HPLC) system 9 (Shimadzu, Kyoto, Japan), equipped with C18 chromatography column with bound residual silane groups (Phenomenex, model 00F-4435-E0) and a UV-Vis detector with a DAD photodiodes array (model SPD-M20A, Shimadzu). The tests were carried out at 45 °C and under isocratic flow conditions of 0.3 mL·min^−1^ and volume composition of the mobile phase of 70% acetonitrile, 29.5% water, and 0.5% orthophosphoric acid. Qualitative and quantitative analysis was performed based on previously made measurements of relevant substance standards [40] and using the method of an external calibration curve.

Phenol removal percentage was calculated from the equation:

(1) $$D\%=\frac{C_{o}-C_{n}}{C_{o}}\cdot 100\%$$

where: C_o_—phenol initial concentration (mg·dm^−3^) and C_n_—phenol concentration during photodegradation (mg·dm^−3^).

Rate constant k was determined from ln(C_o_/C_n_) against t plot where C_o_ and C_n_ are phenol concentrations (mg·dm^−3^) and t is degradation time (min). Rate constant k is equal to the directional coefficient “a” of the plot.

In order to evaluate the stability of obtained photocatalysts, three 3-hours-long subsequent cycles of phenol under UV-Vis light with use of the most active defective TBT-HIO3_50 sample were performed. After each cycle, photocatalyst was separated from the suspension with use of a syringe filter and use in next cycle without additional treatment.

The effect of charge carrier scavengers was examined by addition into phenol solution 1 cm^3^ of 500 mg·dm^−3^ of tert-butyl alcohol (t-BuOH), benzoquinone (BQ) ammonium oxalate (AO), and silver nitrate (SN).

## 3. Results and Discussion

### 3.1. The Influence of Oxidizing Conditions on Defective TiO_2_ Properties

As the first step, a series of three defective TiO_2_ photocatalysts were obtained by the hydrothermal method. The physicochemical characteristic of the obtained samples, including BET surface area with pore volume, bandgap (Eg) and their images, compared with TiO_2_–TBT photocatalyst, are presented in Table 2.

**Figure 1:**
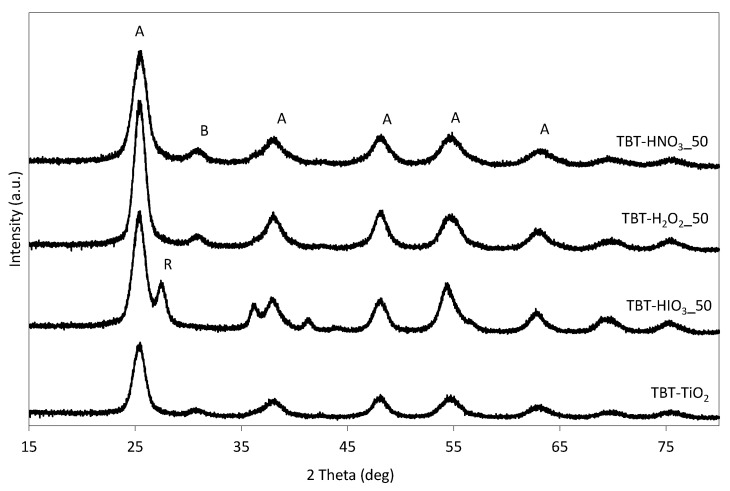
XRD patterns for defective TiO_2_ photocatalysts (A—anatase, B—brookite, and R—rutile).

The XRD patterns for the as-obtained photocatalyst series are presented in Figure 1, while detailed crystalline phases characteristic is given in Table 3. For pure TiO_2_–TBT sample, 95.5% of the crystalline phase of anatase, with the most intense peak at 25° 2θ was observed ([101], ICDD’s card No. 7206075). After introducing to hydrothermal synthesis the oxidizing agent, the percentage of anatase decreased in favor of other titania polymorphs: brookite ([211], with the main peak at 31° 2θ, ICDD’s card No. 9004138), and rutile ([110], with the main peak at 27° 2θ, ICDD’s card No. 9004141). According to the previous study of Gamboa and Pasquevich [41], the presence of halogen ions (chlorine, iodine, and bromine) affects rutile formation even below the anatase to rutile transition (ART) temperature [42]. 

Nonetheless, the anatase crystallite size was about 5–6 nm. Changes in the crystalline phases did not affect the BET surface area, which remained in the range of 166–198 m^2^·g^−1^ for TBT-HIO_3__50 and TBT-HNO_3__50, respectively.

**Figure 2:**
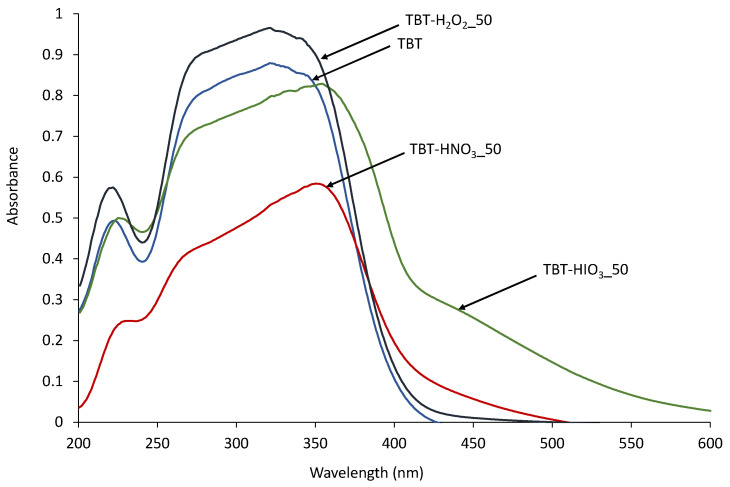
UV-Vis diffuse spectra for pure and defective TiO_2_ obtained using different oxidants.

For optical absorption properties studies of the obtained defective TiO_2_, DR/UV-Vis spectroscopy analyses were performed, and the results are presented in Figure 2. Comparing to pure TBT-TiO_2_, the samples obtained in the oxidative environment had absorption spectra shifted towards the visible light due to the creation of crystalline defects. After recalculation of spectra into the Kubelka–Munk function, the Tauc transformation was used for bandgap energy determination, and its values are presented in Table 2. For TBT-HNO_3__50 and TBT-H_2_O_2__50, the bandgap energies were comparable to pure TBT-TiO_2_ bandgap energy and equaled 3.05 and 3.1 eV, respectively. The slightly smaller value of 2.9 eV was reported for TBT-HIO_3__50 photocatalyst. It could also be caused by a 30% rutile content in the crystal structure of the photocatalyst [43].

**Figure 3:**
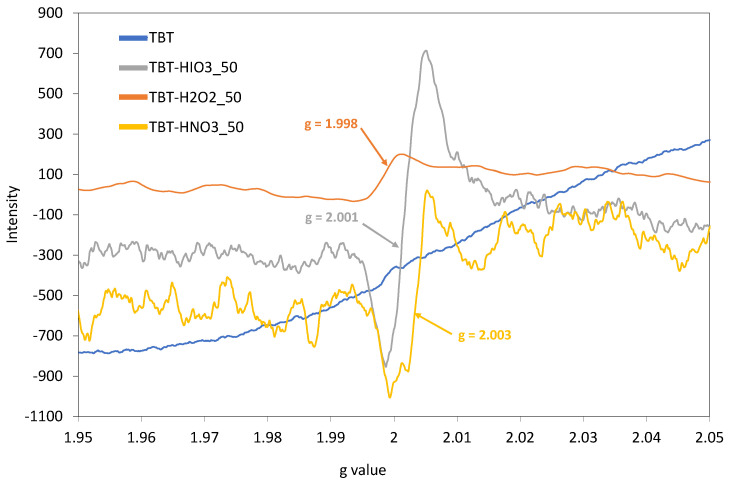
The EPR spectra recorded in the room temperature for defective TiO_2_ photocatalysts obtained in different oxidative environments, compared with pure TBT-TiO_2_ sample (blue line).

For the direct confirmation of intrinsic defects formation in obtained titanium(IV) oxide photocatalysts, EPR analyses were performed, and the results are presented as the signal intensity against the g value graph (Figure 3). The Lande factor (g) was calculated from the equation:

(2) $$g=\frac{h\cdot f}{m_{B}\cdot B}$$

where: g—Lande factor (a.u.); h—Planck’s constant (6.62 × 10^−34^; J·s); f—frequency (Hz); mB—Bohr magneton (9.2740154 × 10^−24^; J·T^−1^); and B—magnetic field induction (T).

For defective TiO_2_ samples, an intense signal was noticed in the range of g from 1.998 to 2.003 for TBT-H_2_O_2__50 and TBT-HIO_3__50 samples, respectively. According to the literature, it could be attributed to titanium vacancies (V_Ti_) in titanium(IV) oxide structure [34,44,45]. This signal was not observed for TBT-TiO_2_ sample. Moreover, there were no signals in the range of g = 1.960–1.990 and above 2.020, suggesting the absence of Ti^3+^ defects as well as oxygen vacancies [45,46].

**Figure 4:**
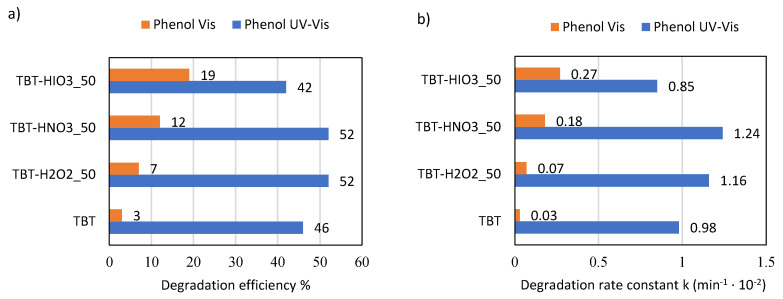
Efficiency of phenol degradation in UV-Vis and Vis light for TBT-TiO_2_ and defective TiO_2_ photocatalysts, presented as % of degradation (**a**) and rate constant k (**b**).

The photocatalytic activity, together with physicochemical properties, are the most important parameters for assessing the semiconductor utility in organic recalcitrant chemicals’ degradation. In this regard, the series of UV-Vis and Vis light degradation reactions of phenol as a model pollutant were performed in the presence of the obtained defective TiO_2_ photocatalysts. The obtained results, presented as the percentage of phenol degradation and degradation rate constant k, are shown in Figure 4a,b.

Comparing to pure TiO_2_ (TBT sample), two defective TiO_2_ photocatalysts, TBT-H_2_O_2__50 and TBT-HNO_3__50, showed higher photocatalytic activity in UV-Vis light (52% after 1 hour of irradiation). In turn, defective TBT-HIO_3__50 obtained in the presence of iodic acid revealed in UV-Vis light decrease of photoactivity, compared with reference TBT-TiO_2_ (42% of phenol degradation). Nonetheless, a different trend was observed under visible light range. TBT-HIO_3__50, for which photoactivity in UV-Vis light was the lowest when irradiated with the wavelength >420 nm, revealed the highest phenol degradation efficiency, equaled to 19%. It results from the synergic effect of anatase and rutile [47], and it is in agreement with the previously described shifting of the absorbance spectrum maximum towards higher wavelengths (see in Figure 2). The TBT-TiO_2_ sample (anatase with a minority of brookite) showed negligible photocatalytic activity in the visible light range [48,49,50].

**Figure 5:**
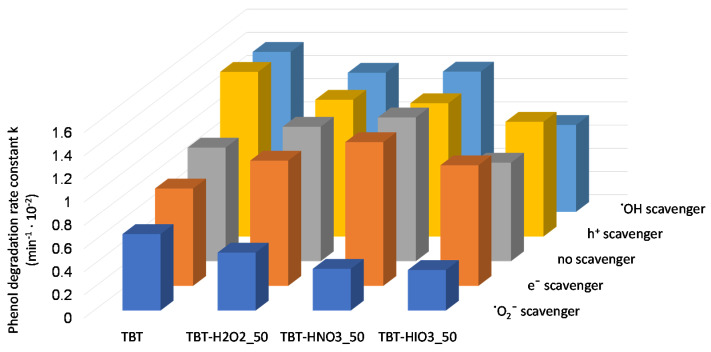
UV-Vis photocatalytic degradation of phenol for defective TiO_2_ photocatalysts in the presence of e^−^, h^+^, ^•^O_2_^−^, and ^•^OH scavengers.

For determining the mechanism of photocatalytic degradation with the use of defective TiO_2_, series of UV-Vis light photoactivity analyses, in the presence of scavengers, were performed. Benzoquinone (BQ), silver nitrate (SN), ammonium oxalate (AO), and tert-butanol (t-BuOH) were used as superoxide radical anions (^•^O_2_^−^), electrons (e^−^), holes (h^+^), and hydroxyl radicals (^•^OH) scavengers, respectively. Obtained results, presented as phenol degradation rate constant k, in comparison to the photodegradation process without scavengers, are presented in Figure 5.

**Figure 6:**
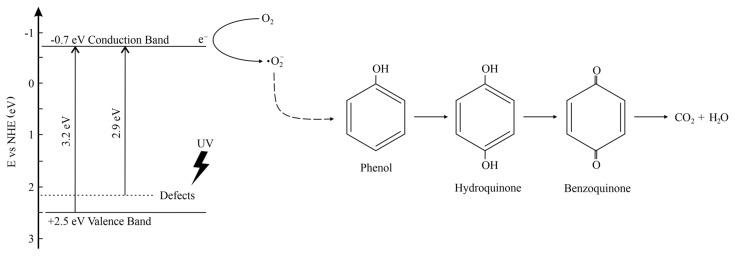
Schematic illustration of phenol degradation mechanism in the presence of defective TBT-HIO_3_ photocatalyst.

The most significant impact on phenol degradation reaction, with the use of defected TiO_2_, revealed superoxide radicals. After introducing to the photocatalyst suspension of BQ, the phenol degradation efficiency decreased significantly. A slight decrease was also observed when SN as an electron trapping agent was used. On the other hand, the addition of AO and t-BuOH did not cause diminishing of phenol degradation rate. Furthermore, for the TBT-TiO_2_ sample, a slight increase in photoactivity was noted after adding to the system scavenger of holes or hydroxyl radicals. It could result from the additional in-situ formation of the reactive species on the photocatalysts’ surface [36]. Based on the study, a schematic mechanism of phenol degradation in the presence of defective TiO_2_ (sample TBT-HIO_3_) was proposed and illustrated in Figure 6.

For pure TiO_2_ the valence band (VB) and conduction band (CB) are located at +2.5 eV and −0.7 eV, respectively (in respect to normal hydrogen electrode NHE) [51]. After hydrothermal treatment in oxidative conditions, titanium defects were created, which led to the narrowing of the bandgap to the value of 2.9 eV. Irradiation of the TBT-HIO_3_ surface with UV-Vis or Vis light caused exciting the electron and, as a result, creating superoxide radicals. Subsequently, their reaction with phenol promotes creating intermediate products, such as hydroquinone (HQ) and benzoquinone (BQ), whose presence was confirmed using HPLC analysis. Benzoquinone and hydroquinone concentration in irradiated solution reached equilibrium due to electron and proton transfer and reversible oxidation/reduction process between these two intermediates. Hydroquinone could also be accumulated during the process due to sequential charge transfer [52]. 

However, after approximately 40 min of continuous irradiation, intermediates concentration started to decrease to more simple and more-quickly oxidizable compounds, consequently leading to complete mineralization. The presented mechanism is in good agreement with the literature [40,53].

### 3.2. The Effect of HIO_3_ Content on Defective TiO_2_ Physicochemical and Photocatalytic Properties

The selected in the previous step HIO_3_ as an oxidant for preparation of defective TiO_2_ was further used in six different quantities (from 0.5 to 100 mol % to TiO_2_) for study the effect of oxidant amount on titanium vacancies formation. General physicochemical characteristics of the obtained defective TiO_2_-HIO_3_ samples, i.e., BET surface area, pore volume, calculated bandgap (Eg), and their images are shown in Table 4.

Based on the obtained results, it was found that changing of the HIO_3_ concentration does not significantly affect the BET surface area of defective TiO_2_-HIO_3_ photocatalysts. Among the obtained samples, TBT-HIO_3__20 showed the highest specific surface area of 172 m^2^·g^−1^ and the highest total pore volume of 0.0847 cm^3^·g^−1^.

**Figure 7:**
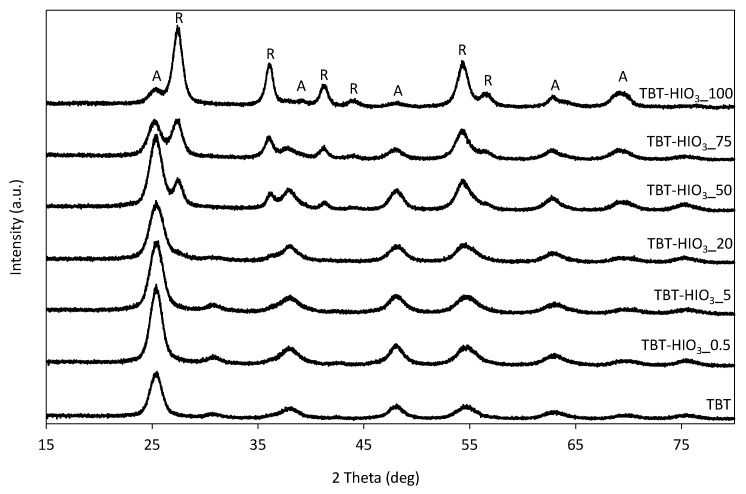
XRD patterns for defective TiO_2_-HIO_3_ photocatalysts (A—anatase, B—brookite, and R—rutile).

The XRD patterns of TBT-TiO_2_ and defective TiO_2_-HIO_3_ obtained with a different dosage of iodic acid are presented in Figure 7. The percentage of phases and the size of crystallites are given in Table 5. All photocatalysts contain anatase in their structure, with the most intense peak at 25° 2θ ([101], ICDD’s card No. 7206075). Among the defective TiO_2_-HIO_3_ photocatalyst series, TBT-HIO_3__20 exhibited the smallest size of anatase crystallites (5.1 nm based on the Scherrer’s formula) and was characterized by the highest anatase phase content of 96.4%. The most stable titanium(IV) oxide polymorphic phase, rutile, occurs when the mol.% of iodic acid taken as an oxidant reached 50 mol%. Simultaneously, the intensity of the primary rutile signal at 27° 2θ ([110], ICDD’s card No. 9004141), increased significantly with the increase of iodic acid dosage for samples TBT-HIO_3__50, TBT-HIO_3__75, and TBT-HIO_3__100. It is known that for an unmodified sample, anatase to rutile transition takes place at temperatures above 600 °C [54]. Obtained TBT-HIO_3_ samples calcination was carried out at 300 °C. On this basis, it could be assumed that the high content of HIO_3_ may disturb the TiO_2_ anatase crystalline structure, therefore promoting the low-temperature formation of rutile. It is in agreement with the study of Hanaor and Sorrell [55], which reported that impurities, dopants, and defects influence anatase to rutile transition (ART) kinetics.

**Figure 8:**
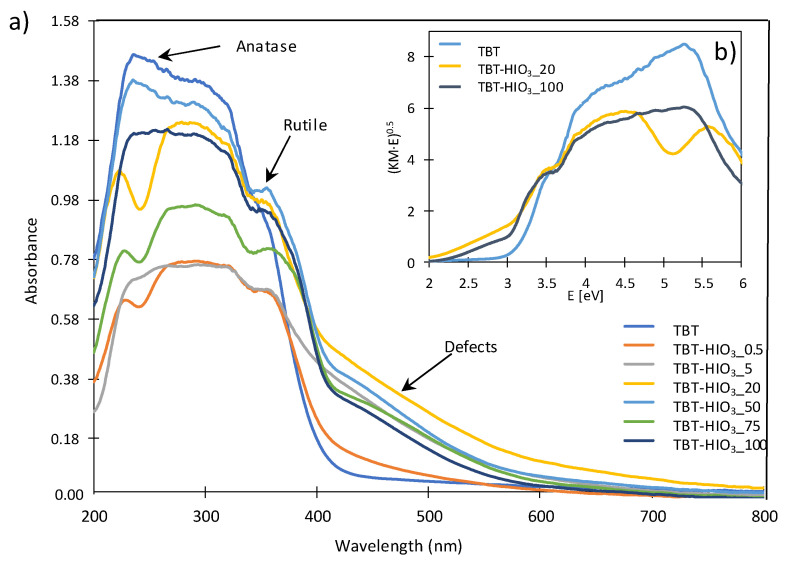
The diffuse reflectance (DR)/UV-Vis spectra for pure TiO_2_ and defective TiO_2_-HIO_3_ photocatalysts (**a**) together with exemplary Tauc transformation (**b**).

The UV/Vis diffusion reflectance spectra of pure TiO_2_ and defective TiO_2_-HIO_3_ obtained with a different dosage of iodic acid are presented in Figure 8a. The pure TiO_2_ absorbs radiation up to 400 nm. For titanium(IV) oxide samples obtained in the presence of HIO_3_ as an oxidant, the absorption edge shifted to the visible region. It corresponds to the yellow colour of these samples and indicates the bandgap narrowing due to changes in electronic structure in TiO_2_. The most significant shift of absorbance maximum was noticed for TBT-HIO_3__50, TBT-HIO_3__75, and TBT-HIO_3__100 photocatalysts. It corresponds well with previously analysed XRD spectra. For iodic acid content of 50 mol% and higher, rutile phase is starting to dominate as a titanium(IV) oxide most stable polymorph, which could also affect absorbance spectra shifting [56]. The energy bandgaps of all samples were calculated from the plot of (Kubelka–Munk·E)^0.5^ versus E, where E is energy equal to hv, as shown in Figure 8b and summarized in Table 4. The sample TBT-HIO_3__20 exhibited the narrowest bandgap of 2.70 eV among the defective TiO_2__HIO_3_ photocatalysts.

**Figure 9:**
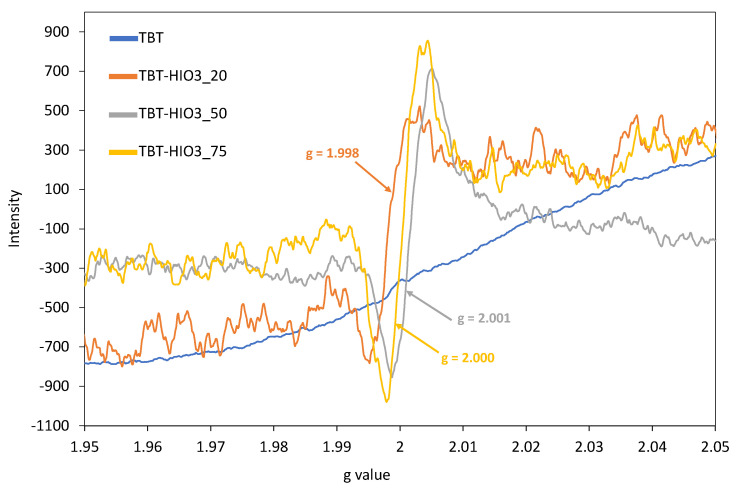
The EPR spectra recorded in the room temperature for selected defective TiO_2_-HIO_3_ photocatalysts, compared with the pure TiO_2_–TBT sample (blue line).

For further confirmation of creating titanium vacancies, EPR analyses for the selected samples (TBT-HIO_3__20, TBT-HIO_3__50, and TBT-HIO_3__75) were performed. The obtained results, compared with spectra for TBT-TiO_2_ are presented in Figure 9.

As it was reported in the previous subsection, for defective TiO_2_ photocatalysts obtained in a different oxidative environment, the intense signal attributed to titanium vacancies (V_Ti_) appeared in the range of g from 1.998 to 2.001 for TBT-HIO_3__20 and TBT-HIO_3__50 samples, respectively. No additional signals were detected. It is also worth noting that the V_Ti_ signal increased with the increase of the iodic acid mol% used for the synthesis of defective TiO_2_. It could suggest that more intrinsic defects are formed after oxidant concentration increase. Moreover, the presented trend was inversely proportional to the observed light absorbance spectra in the range of 400–500 nm (see in Figure 8). For the TBT-HIO_3__20 photocatalyst, visible light absorption was the highest, while for TBT-HIO_3__75, the lowest, which indicated that too high concentration of defects could also have a negative impact on TiO_2_ photocatalytic activity. Titanium vacancies formation was also analyzed by Li et al. [57] and Ma et al. [58]. Obtained hydroxyfluorinated and lithium intercalated defected TiO_2_-based photocatalysts were characterized by cationic vacancies, which successfully could work in the electrochemical applications.

**Figure 10:**
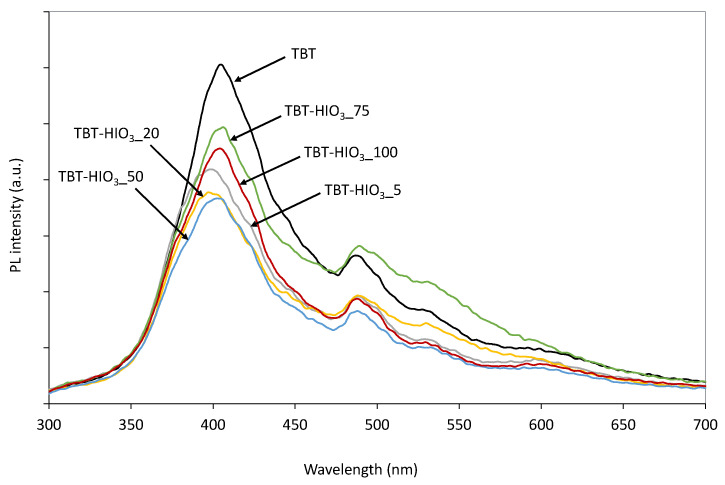
Photoluminescence (PL) spectra for defective TiO_2_-HIO_3_ samples.

The photoluminescence spectra of irradiated semiconductor materials give information on electron-hole recombination properties. Figure 10 shows normalized PL spectra of the pure TBT-TiO_2_ as well as defective TBT-HIO_3__5, TBT-HIO_3__20, TBT-HIO_3__50, TBT-HIO_3__75, and TBT-HIO_3__100 photocatalyst samples. The excitation was carried out at 250 nm at a room temperature. The PL emission of maximum intensity in the high-energy region was observed at 400 nm for all samples, which was equal to 3.10 eV. It corresponds to indirect band-to-band recombination across the bandgap [59]. Other emission peaks in the visible light region were detected at 485 nm (2.56 eV) and 530 nm (2.24 eV). The emission in the 380–700 nm range could be assigned to the transition of electrons from the defect states to the valence band of titanium(IV) oxide [60] as well as trapped holes [59]. The emission in the blue region at 480 nm is related to indirect recombination via defects [60].

**Figure 11:**
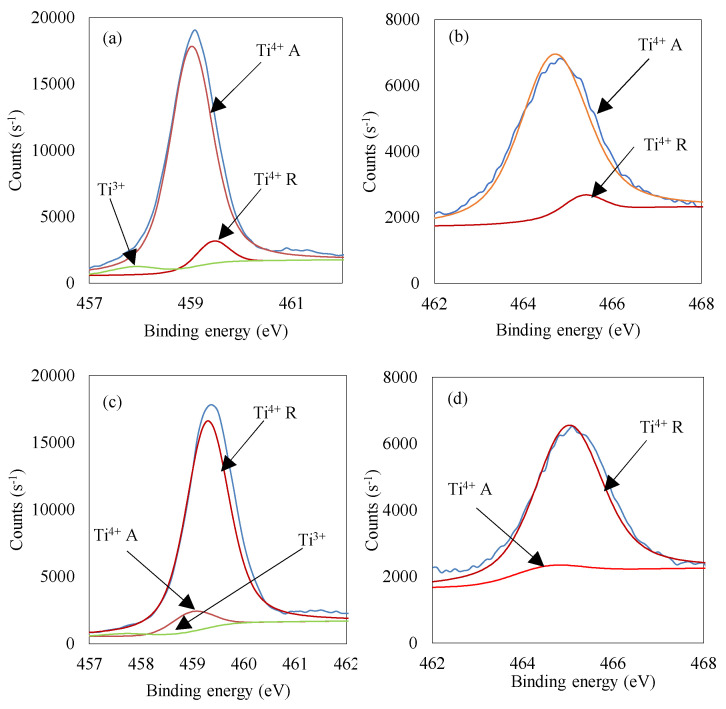
Deconvolution of X-ray photoelectron spectroscopy (XPS) spectra for Ti 2p_3/2_ and 2p_1/2_ for TBT-HIO_3__20 (**a**,**b**) and TBT-HIO_3__100 (**c**,**d**).

In order to evaluate the surface properties and the state of elements, the XPS analyses were performed. The obtained results for the selected samples are presented in Figure 11a–d and in Table 6.

The Ti 2p spectrum could be deconvoluted into two components at 459 eV and 465 eV binding energies that refer to Ti 2p_3/2_ and 2p_1/2_, respectively. Ti 2p_3/2_ after deconvolution could be divided into 459.0 eV and 459.5 eV peaks and identified as Ti^4+^, resulting from the presence of anatase and rutile, respectively. For sample TBT-HIO_3__20 a trace quantity (0.9 at.%) of Ti^3+^ was observed, which could be assigned to oxygen vacancies [61]. However, apart from this sample, there was no Ti^3+^ signal observed, suggesting the lack of reduced form of titanium as well as oxygen vacancies. The presented XPS titanium peaks corresponding to both anatase and rutile correlate with XRD analysis. Together with the increase of HIO_3_ oxidant amount used for the synthesis, the rutile content increase, which is also clearly visible in the presented spectra. The Ti/O ratio for all analyzed photocatalysts was equaled to 0.38. It suggests no surface and state of elements change between TBT-HIO_3_ samples.

**Figure 12:**
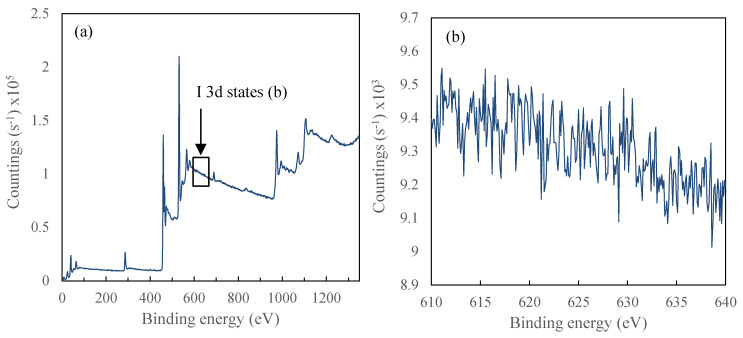
XPS analyses spectra for TBT-HIO_3__20 sample (**a**) with the I 3d states binding energy enlargement (**b**).

For final evaluation, since the synthesized photocatalysts do not have admixtures but their color as well as physicochemical and photocatalytic properties arise from created intrinsic defects the region I 3d was analyzed to check the presence of iodine species in the obtained samples. The results are presented in Figure 12.

As mentioned in the literature [62,63,64] I 3d states are in the range of 620–635 eV. However, as it could be seen in Figure 12b, there is no peak, which could be assigned to I 3d states. In this regard, for TBT-HIO_3_ samples changes in physicochemical and, what is the most important, photochemical properties are caused by intrinsic defects, not titania doping with impurities.

In order to evaluate morphological differences among the obtained defective TiO_2_ photocatalysts, the SEM analysis for selected samples was conducted, and the results are presented in Figure S1 in the Supplementary Materials. It was found that both samples are formed from aggregated particles. However, it is noticeable that aggregates of TBT-HIO_3__50 had a much smaller size, comparing to the TiO_2_-TBT photocatalyst, although no differences were determined in crystalline sizes or the BET specific surface area.

**Figure 13:**
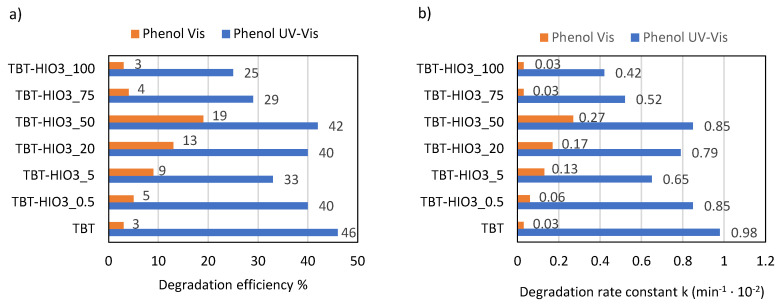
Efficiency of phenol degradation in UV-Vis and Vis light for defective TiO_2_-HIO_3_ photocatalysts, presented as % of degradation (**a**) and rate constant k (**b**).

**Figure 14:**
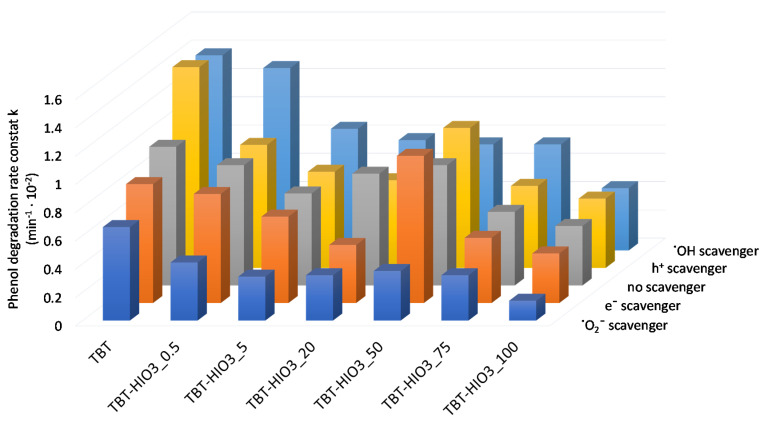
UV-Vis photocatalytic degradation of phenol for TiO_2_-HIO_3_ photocatalyst in the presence of e^−^, h^+^, ^•^O_2_^−^, and ^•^OH scavengers.

The photocatalytic activity of defective TiO_2_-HIO_3_ samples was evaluated in the phenol degradation reaction, both in UV-Vis and Vis (λ > 420 nm) light. Simultaneously, the effect of e^−^, h^+^, ^•^O_2_^−^, and ^•^OH scavengers’ presence on photoactivity was studied. The results, presented as the efficiency of phenol removal (%) as well as phenol degradation rate constant k are presented in Figure 13a,b and Figure 14.

The best photocatalytic activity was obtained for the defective TBT-HIO_3__50 sample. After 60 min of irradiation, about 42% of phenol was degraded in UV-Vis and 19% in Vis light. It may also be noticed a characteristic normal distribution of the obtained results, where maximum falls on 50 mol% of iodic acid. Both smaller and higher concentrations of oxidant used in hydrothermal synthesis process caused a decrease in obtained TBT-HIO_3_ photoactivity. 

The obtained photodegradation efficiency results correlate well with photoluminescence (PL) spectra, UV-Vis spectra, and EPR spectra analyses. From the three analyzed samples (TBT-HIO_3__20, TBT-HIO_3__50, and TBT-HIO_3__75), the most intense EPR signal was assigned to defective TBT-HIO_3__75, where the highest concentration of oxidant (75 mol%) was used. From the PL spectra analysis, the TBT-HIO_3__75 sample showed the highest intensity among analyzed photocatalysts, which indicated the highest electron-hole recombination as well as the lowest phenol degradation efficiency. It could suggest that too high of a concentration of defects in the TiO_2_ structure could significantly decrease the photocatalytic activity of the defective material. The presented results also correlate with the crystalline structure of the obtained materials. With the increase of the HIO_3_ concentration, the rutile content was increased (up to 80% for the TBT-HIO_3__75 photocatalyst). According to the literature, too high rutile concentration could also be responsible for decreasing of the TiO_2_ photocatalytic activity [65].

The addition of BQ as an ^•^O_2_^−^ scavenger caused a significant reduction of photoactivity of all obtained TBT-HIO_3_ photocatalysts, regardless of the used HIO_3_ concentration for their synthesis. It indicated that superoxide radical anions are the most crucial reactive oxygen species in the photocatalytic reaction with the use of defective TBT-HIO_3_ samples. After introducing to the photoreactive SN, AO, and t-BuOH, the changes of the phenol degradation rate constant k, comparing to the process without scavenger, were negligible.

The physicochemical and surface properties of the most active defective TiO_2_ photocatalyst (TBT-HIO_3__50) was analyzed before and after 1 hour of phenol degradation process in the presence of UV-Vis irradiation to confirm the photocatalyst stability. The obtained results are presented in Figures S2–S4 and in Table S1 in the Supplementary Materials. The additional XPS as well as XRD analysis showed, that after 1 hour of degradation process the physicochemical properties, e.g., crystalline size and surface composition did not change. Moreover, both TBT-HiO_3__50 samples showed also a similar FTiR spectra (see in Figure S3) with a broad band at 3450–3050 cm^−1^ attributed to the stretching mode of the hydroxyl group on the TiO_2_ surface. The Ti-O bending mode and deformative vibration of the Ti-OH stretching mode may be observed at 498-463 cm^−1^ and 1629 cm^−1^ respectively. The band at 1629 cm^−1^ may be attributed to water adsorbed on the TiO_2_ surface.

**Figure 15:**
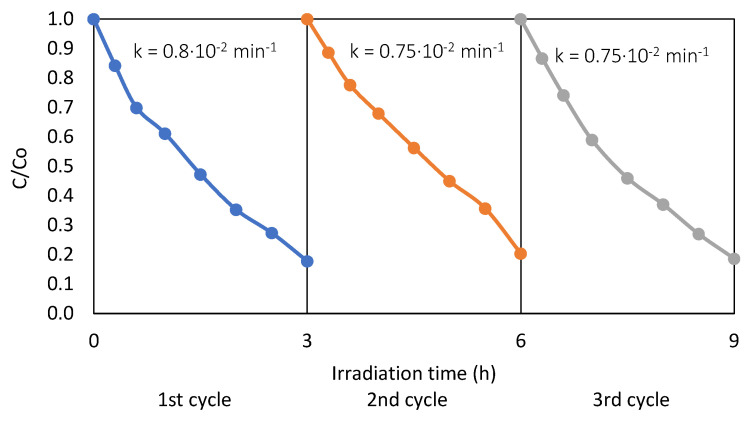
Efficiency of UV-Vis phenol degradation in the presence of a defective TBT-HIO_3__50 photocatalyst measured in the three subsequent cycles.

Final stability and reusability test of defective TBT-HIO_3__50 photocatalyst was performed in three 3-hours-long subsequent cycles of phenol degradation under UV-Vis light. The obtained results are presented in Figure 15.

After 9 h of irradiation, the percentage of degraded phenol was 80%, which is almost equal to photodegradation efficiency after 3 h (82%). A slight drop in the rate constant k could be seen (from k = 0.8·× 10^−2^ min^−1^ after first cycle to k = 0.75·× 10^−2^ min^−1^ after the second and the third cycle). However, the analysed photocatalyst still revealed good stability and reusability.

### 3.3. The Effect of Thermal Treatment on Defective TiO_2_-HIO_3_ Physicochemical and Photocatalytic Properties

Further, the investigation on defective photocatalysts concerned with the thermal stability of TiO_2_-HIO_3_ samples was undertaken. A series of five TiO_2_-HIO_3_ photocatalysts, calcined in different temperatures from 300 to 1000 °C was obtained. As a reference, as characterized earlier, the TBT-HIO_3__20 photocatalyst was used, named as TBT-HIO_3__20_300, as it was calcined at 300 °C. General physicochemical characteristics of the obtained defective TiO_2_-HIO_3__T samples, i.e., BET surface area, pore volume, calculated bandgap (Eg), and their images are shown in Table 7.

The addition of HIO_3_ to the reaction environment had a negligible effect on changing the BET surface area as well as particles and crystallites sizes. Nonetheless, increasing the calcination temperature by 100 °C led to a 50% surface area decreasing (from 172 to 88 m^2^·g^−1^ for TBT-HIO_3__20_300 and TBT-HIO_3__20_400, respectively). Further increasing of the thermal treatment up to 1000 °C caused the decrease of the BET surface area to 0.4 m^2^·g^−1^.

**Figure 16:**
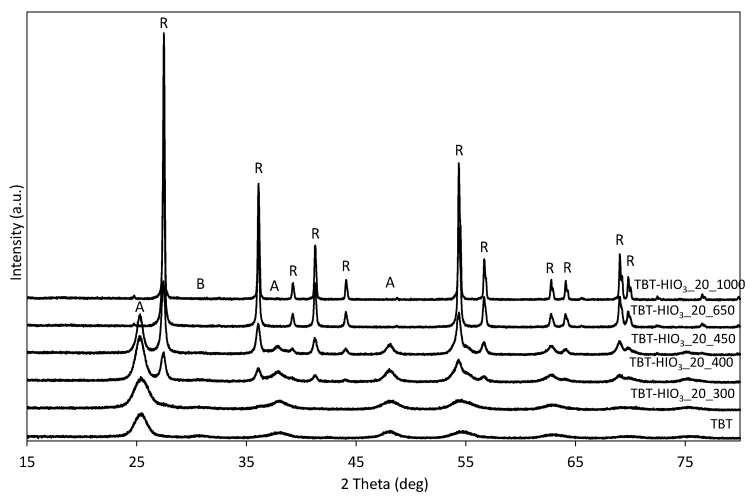
XRD patterns for defective TiO_2_-HIO_3__T photocatalysts (A—anatase, B—brookite, and R—rutile).

Changes in the BET surface area correlate well with differences noted on the XRD patterns for TBT-HIO_3__T samples, presented in Figure 16. The higher the calcination temperature, the more intense the XRD diffraction peaks, which resulted from the increase in photocatalysts crystallinity [66]. Other changes concern crystallites growth (for anatase: from 5 to 12 nm TBT-HIO_3__20_300 and TBT-HIO_3__20_450, respectively and for rutile: from 17 to 53.5 nm for TBT-HIO_3__20_400 and TBT-HIO_3__20_1000, respectively) as well as the anatase to rutile phase transition. No rutile phase was present in TBT-HIO_3__20_300 photocatalyst, while its content increased rapidly as the calcination temperature increased until it reached 100% at T = 650 °C. As mentioned before, the anatase to rutile transition takes place in about 600 °C. However, the introduction to crystal structure various types of defects promotes this transformation at lower temperatures [67].

The detailed information about crystallite sizes and phase contents presented with standard deviation are given in Table 8.

**Figure 17:**
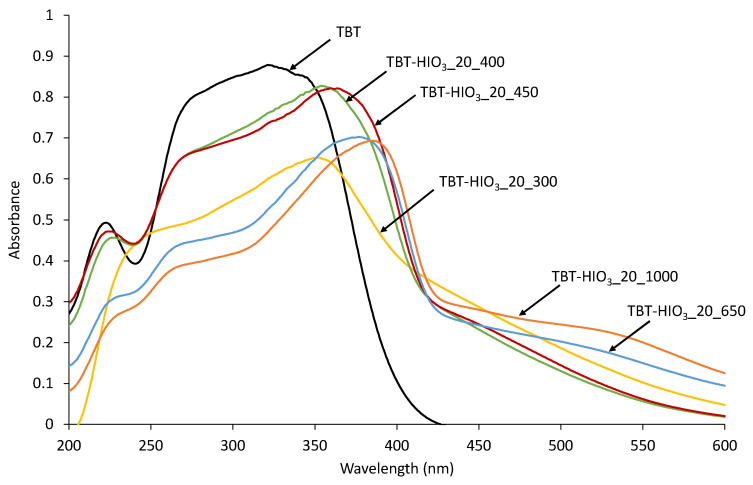
UV-Vis diffuse spectra for pure TiO_2_ and defective TiO_2_-HIO_3__20_T photocatalysts calcined in different temperatures.

Shifting of the absorption maximum on DR/UV-Vis spectra for defective TiO_2_-HIO_3__T samples (Figure 17) was mostly related to the anatase to rutile phase transition. It was mentioned by Valencia et al. [68] that anatase bandgap is equal to 3.23 eV, while rutile from 3.06 to 3.10 eV. From Tauc transformation, bandgap values forr TiO_2_-HIO_3__T photocatalysts were calculated and are in the range from 2.7 to 2.9 eV. Despite the changes in photocatalysts phase contents a slight decrease in bandgap value, in response to TiO_2_, could be caused by defects formation in the crystal structure [69].

**Figure 18:**
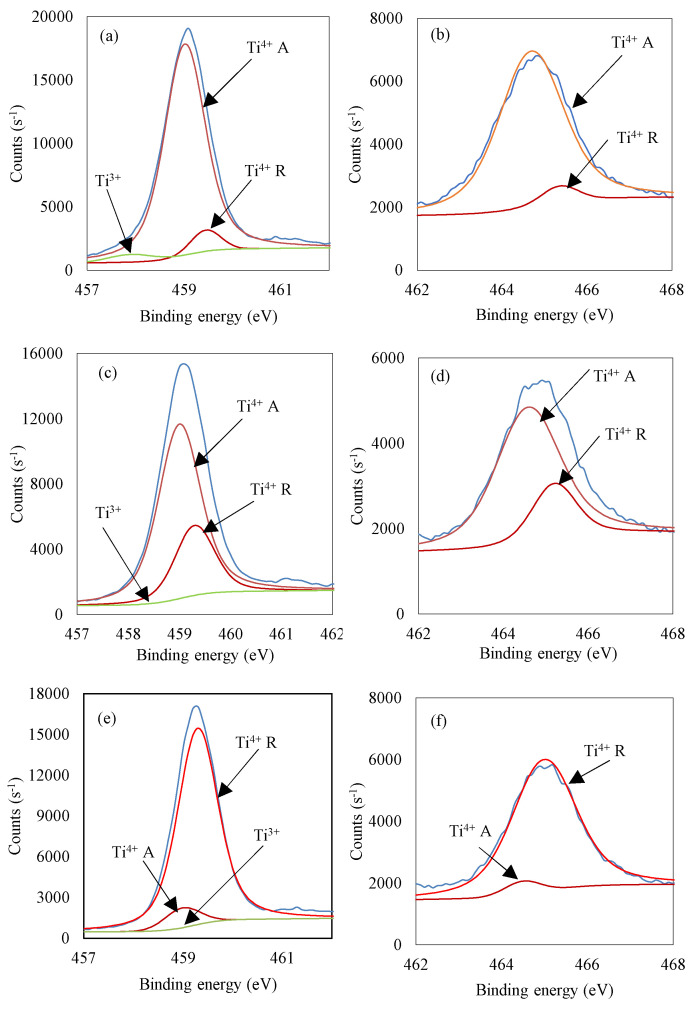
Deconvolution of X-ray photoelectron spectroscopy (XPS) spectra for Ti 2p_3/2_ and 2p_1/2_ for TBT-HIO_3__20_300 (**a**,**b**), TBT-HIO_3__20_400 (**c**,**d**) and TBT-HIO_3__20_450 (**e**,**f**).

Similarly to TBT-HIO_3_ photocatalysts, TBT-HIO_3__20_T samples’ surface properties, as well as the state of elements, were analyzed using XPS analysis. The obtained results are presented in Figure 18a–f and Table 9.

States of elements for thermally treated TBT-HIO_3__20_T are identical as for previously described TBT-HIO_3_ samples. Oxygen vacancies are not detected, except TBT-HIO_3__20_300 photocatalyst with 0.9 at.% of Ti^3+^ form. Observed deconvoluted spectra for Ti 2p_3/2_ and 2p_1/2_ corresponded well with XRD analysis and showed the anatase–rutile transition. 

**Figure 19:**
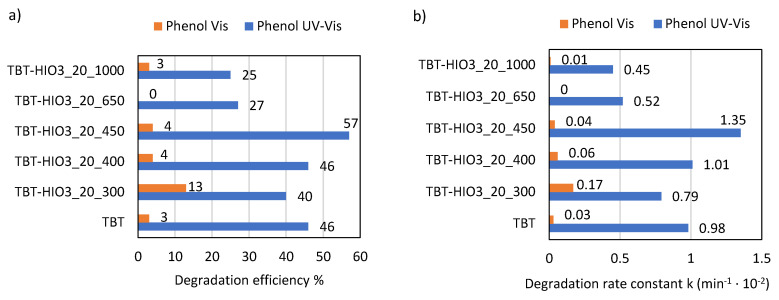
Efficiency of phenol degradation in UV-Vis and Vis light for defective TiO_2_-HIO_3__20_T photocatalysts, presented as % of degradation (**a**) and rate constant k (**b**).

**Figure 20:**
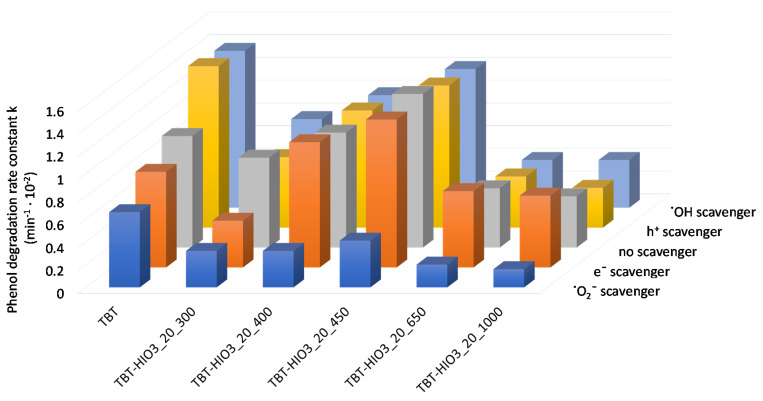
UV-Vis photocatalytic degradation of phenol for TiO_2_-HIO_3__20_T photocatalysts in the presence of e^−^, h^+^, ^•^O_2_^−^, and ^•^OH scavengers.

For the final evaluation, photocatalytic activity tests for TiO_2_-HIO_3__20_T samples in the phenol degradation reaction, both in UV-Vis and Vis (λ > 420 nm) light, were performed. Simultaneously, the effect of e^−^, h^+^, ^•^O_2_^−^, and ^•^OH scavengers’ presence on photoactivity was studied. Results, presented as phenol removal in % as well as phenol degradation rate constant k, are presented in Figure 19a,b and Figure 20.

According to the literature, the optimum calcination temperature for iodine-doped photocatalysts is between 300 and 400 °C, with the temperature of 300 °C preferred when potassium iodide is used as an iodine precursor, while 400 °C when iodic acid is applied as a precursor [70,71]. Above these temperatures, the photoactivity of prepared materials decreased significantly. Nonetheless, for TBT-HIO_3_ defective photocatalysts, where iodic acid was used as an oxidative environment for titanium vacancies generation, the highest efficiency in phenol degradation reaction was noticed for sample calcined in 450 °C (57% of phenol removal after 1 h of UV-Vis light irradiation). Moreover, the yellow color of the sample was maintained even after calcination at 1000 °C (see in Table 7). It suggests that the obtained intrinsic defects in the crystal structure of TiO_2_ were stable even in higher calcination temperatures. A slightly different situation was observed when photoactivity tests were carried out in the visible light. Apart from TBT-HIO_3__20_300 photocatalyst, all samples showed negligible efficiency in phenol degradation reaction. However, this could be caused by increasing rutile phase content in the photocatalyst structure [72].

For TiO_2_-HIO_3__20_T photocatalysts, there was no difference in mechanistic studies as compared to the previously discussed defected TiO_2_ series. The superoxide radical anions remained the most crucial for the phenol degradation reaction, while the addition of ammonium oxalate, tert-butyl alcohol, and silver nitrate as scavengers did not affect the photoactivity.

## 4. Conclusions

Hydrothermal treatment in the presence of an oxidative environment led to titanium vacancies generation in the structure of TiO_2_. Created intrinsic defects caused yellow coloration of titania, while at the same time, absorption of semiconductor was shifted to visible light as well as bandgap was reduced to 2.9 eV. Among studied oxidants, defective TiO_2_ samples obtained in the presence of iodic acid were characterized by the highest phenol degradation efficiency in visible light. In-depth analysis, including EPR and XPS measurements, confirmed that increasing in photoactivity, compared to pure material, is directly caused by defects, not by doping. Further analysis regarding the optimum amount of HIO_3_ as well as the thermal stability of synthesized defected TiO_2_-HIO_3_ photocatalysts, showed that from 20 to 50 mol% of oxidant added to hydrothermal reaction is capable of creating material with a great photoactivity and no loss in photoactivity up to 450 °C.

## Figures and Tables

**Table 1:** The oxidant concentration used for preparation of the defective TiO_2_ photocatalyst.

Sample	Oxidant Concentration (mol%)	Mass of Added Oxidant (g)
TiO_2_-TBT	0	0
TBT-HIO_3__0.5	0.5	0.026
TBT-HIO_3__5	5	0.258
TBT-HIO_3__20	20	1.032
TBT-HIO_3__50	50	2.579
TBT-HIO_3__75	75	3.869
TBT-HIO_3__100	100	5.159
TBT-HNO_3__50	50	0.948
TBT-H_2_O_2__50	50	1.65

**Table 2:** Physicochemical characteristic of the obtained defective TiO_2_ samples.

Sample	BET (m^2^·g^−1^)	V Pores (cm^3^·g^−1^)	Eg (eV)	Photo
TiO_2_-TBT	169	0.0836	3.2	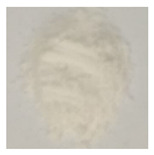
TBT-HIO_3__50	166	0.0818	2.9	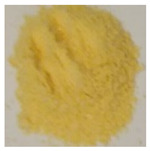
TBT-HNO_3__50	198	0.0966	3.05	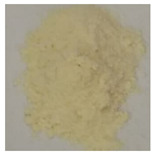
TBT-H_2_O_2__50	174	0.0858	3.1	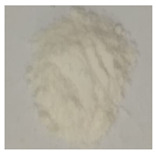

**Table 3:** Crystalline phases characteristic for obtained defective TiO_2_.

Sample	Crystalline Size and Phase Content					
	Anatase		Rutile		Brookite	
	Size (nm)	Phase Content (wt %)	Size (nm)	Phase Content (wt %)	Size (nm)	Phase Content (wt %)
TBT	5.97 ± 0.04	95.5 ± 1	-	-	6.1 ± 0.5	4.5 ± 0.9
TBT-HIO_3__50	5.70 ± 0.04	68 ± 3	9.08 ± 0.17	32 ± 17	-	-
TBT-HNO_3__50	5.07 ± 0.03	83 ± 10	-	-	4.6 ± 0.3	17 ± 2
TBT-H_2_O_2__50	5.69 ± 0.04	75.5 ± 5	-	-	5.7 ± 0.3	24.5 ± 1.5

**Table 4:** Physicochemical characteristic of the obtained defective TiO_2_-HIO_3_ samples.

Sample	BET (m^2^·g^−1^)	V Pores (cm^3^·g^−1^)	Eg (ev)	Photo
TBT	169	0.0836	3.2	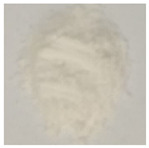
TBT-HIO_3__0.5	155	0.0764	3.0	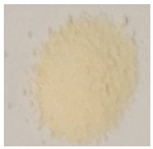
TBT-HIO_3__5	153	0.0754	2.8	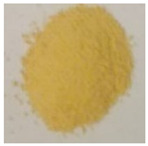
TBT-HIO_3__20	172	0.0847	2.7	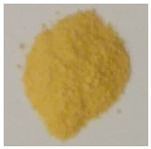
TBT-HIO_3__50	166	0.0818	2.9	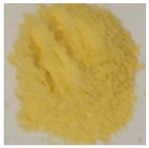
TBT-HIO_3__75	167	0.0826	2.9	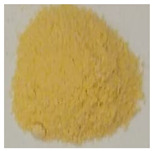
TBT-HIO_3__100	146	0.0726	3.0	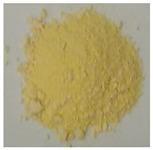

**Table 5:** Crystalline phases characteristic for the obtained defective TiO_2_-HIO_3_ samples.

Sample	Crystalline Size and Phase Content					
	Anatase		Rutile		Brookite	
	Size (nm)	Phase Content (wt%)	Size (nm)	Phase Content (wt%)	Size (nm)	Phase Content (wt%)
TBT	5.97 ± 0.04	95.5 ± 1	-	-	6.1 ± 0.3	4.5 ± 0.9
TBT-HIO_3__0.5	6.09 ± 0.03	86 ± 1	-	-	5.50 ± 0.19	14 ± 1.5
TBT-HIO_3__5	5.43 ± 0.03	89 ± 0.5	-	-	5.2 ± 0.2	11 ± 1
TBT-HIO_3__20	5.14 ± 0.03	96 ± 1	-	-	4.0 ± 0.6	3.5 ± 0.5
TBT-HIO_3__50	5.70 ± 0.04	68 ± 3.5	9.08 ± 0.17	32 ± 17	-	-
TBT-HIO_3__75	5.67 ± 0.05	20.5 ± 3.5	6.57 ± 0.09	7± 1.8	-	-
TBT-HIO_3__100	6.3 ± 0.2	15 ± 3.5	7.45 ± 0.06	85 ± 1	-	-

**Table 6:** Fraction of oxidation states of Ti as well as surface composition of the selected defected TBT-HIO_3_ photocatalysts determined by X-ray photoelectron spectroscopy analysis.

Photocatalyst	Ti 2p_3/2_ (%)		O 1s (%)
	Ti^4+^	Ti^3+^	
TBT-HIO_3__20	27.56	0.9	71.54
TBT-HIO_3__100	27.47	0	72.54

**Table 7:** Physicochemical characteristic of the obtained defective TiO_2_-HIO_3__T samples.

Sample	BET (m^2^·g^−1^)	V Pores (cm^3^·g^−1^)	Eg (ev)	Photo
TBT	169	0.0836	3.2	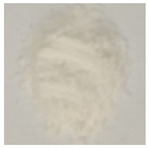
TBT-HIO_3__20_300	172	0.0847	2.7	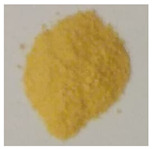
TBT-HIO_3__20_400	88	0.0432	2.85	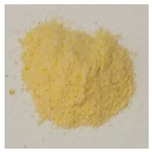
TBT-HIO_3__20_450	48	0.0236	2.9	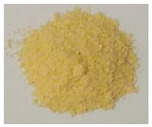
TBT-HIO_3__20_650	0.7	0.0003	2.9	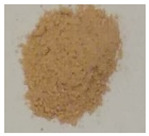
TBT-HIO_3__20_1000	0.4	0.0002	2.8	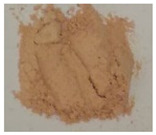

**Table 8:** Crystalline phases characteristic for the obtained defective TiO_2_-HIO_3__T samples.

Sample	Crystalline Size and Phase Content					
	Anatase		Rutile		Brookite	
	Size (nm)	Phase Content (wt%)	Size (nm)	Phase Content (wt%)	Size (nm)	Phase Content (wt%)
TBT	5.97 ± 0.04	95.5 ± 1	-	-	6.1 ± 0.3	4.5 ± 1
TBT-HIO_3__20_300	5.14 ± 0.03	96 ± 1	-	-	4.0 ± 0.6	4 ± 0.5
TBT-HIO_3__20_400	8.34 ± 0.05	70.5 ± 0.5	17.4 ± 0.2	29.5 ± 0.5	-	-
TBT-HIO_3__20_450	12.13 ± 0.09	40 ± 0.5	22.51 ± 0.19	60.5 ± 0.5	-	-
TBT-HIO_3__20_650	-	-	40.3 ± 0.3	100 ± 0.5	-	-
TBT-HIO_3__20_1000	-	-	53.5 ± 0.3	100 ± 0.5	-	-

**Table 9:** Fraction of oxidation states of Ti as well as surface composition of selected defected TBT-HIO_3__20_T photocatalysts determined by X-ray photoelectron spectroscopy (XPS).

Photocatalyst	Ti 2p_3/2_ (%)		O 1s (%)
	Ti^4+^	Ti^3+^	
TBT-HIO_3__20_300	27.56	0.9	71.54
TBT-HIO_3__20_400	27.99	0	72.01
TBT-HIO_3__20_450	28.43	0	71.58

## Supplementary Materials

The following are available online at https://www.mdpi.com/1996-1944/13/12/2763/s1, Figure S1: SEM analysis of pure TiO_2_-TBT (**a**,**b**) and defective TBT-HIO3_50 (**c**,**d**) photocatalysts, Figure S2: Deconvolution of X-ray photoelectron spectroscopy (XPS) for Ti 2p3/2 and O 1s for TBT-HIO3_50 before (**a**,**b**) and after phenol photodegradation (**c**,**d**). Figure S3: XRD patterns for defective TBT-HIO3_50 photocatalyst before and after a photocatalytic phenol degradation (A–anatase, R–rutile). Figure S4: Fast-Fourier transformation spectroscopy (FTIR) spectra of defective TBT-HIO3_50 before and after phenol photodegradation. Table S1: Fraction of oxidation states of Ti as well as surface composition of defective TBT-HIO3_50 photocatalyst before and after phenol degradation.
