# Supplementary material for: Preparation and Characterization of Defective TiO2. The Effect of the Reaction Environment on Titanium Vacancies Formation
Source: Materials (Basel). 2020 Jun 18;13(12):2763. doi: 10.3390/ma13122763 (PMC7345791; doi:10.3390/ma13122763)
Supplement: Supplementary file 1 [file materials-13-02763-s001.pdf]

materials

Supplementary Material

Preparation and Characterization of Defective TiO_2. The Effect of the Reaction Environment on Titanium Vacancies Formation.

Zuzanna Bielan [1,*], Szymon Dudziak [1], Agnieszka Sulowska [1], Daniel Pelczarski [2], Jacek Ryl [3] and Anna Zielińska-Jurek [1,*]

[1] Department of Process Engineering and Chemical Technology, Faculty of Chemistry, Gdansk University of Technology (GUT), G. Narutowicza 11/12, 80-233 Gdansk, Poland; sulowska.as@gmail.com (A.S); dudziakszy@gmail.com (S.D.)
[2] Department of Physics of Electronic Phenomena, Faculty of Applied Physics and Mathematics, Gdansk University of Technology (GUT), G. Narutowicza 11/12, 80-233 Gdansk, Poland; daniel.pelczarski@pg.edu.pl
[3] Department of Electrochemistry, Corrosion and Materials Engineering, Faculty of Chemistry, Gdansk University of Technology (GUT), G. Narutowicza 11/12, 80-233 Gdansk, Poland; jacryl@pg.edu.pl
* Correspondence: annjurek@pg.edu.pl (A.Z.-J.), zuzanna.bielan@gmail.com (Z.B.)

Received: 13 May 2020; Accepted: 16 June 2020; Published: 18 June 2020

In the Figure S1 are presented SEM images for pure TiO_2-TBT (a,b) and TBT-HIO_3_50 (c,d).

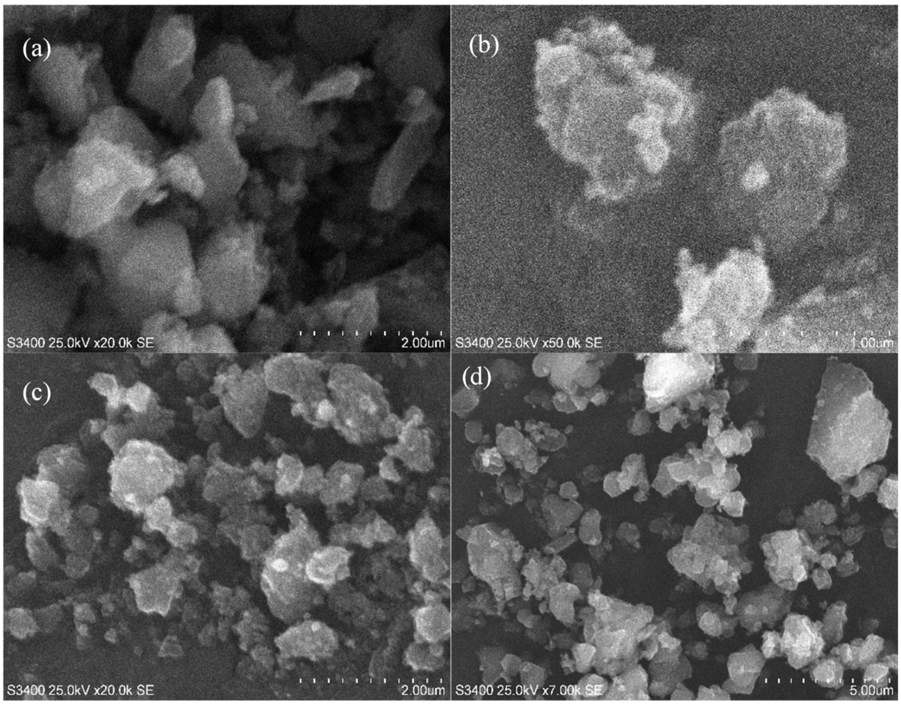

Figure S1. SEM analysis of pure TiO_2-TBT (**a,b**) and defective TBT-HIO_3_50 (**c,d**) photocatalysts.

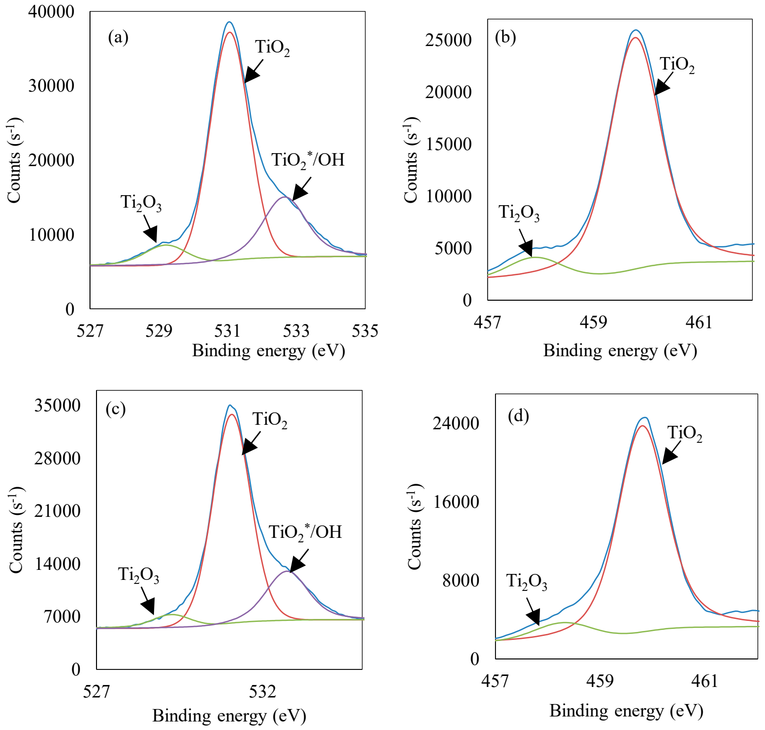

Figure S2. Deconvolution of X-ray photoelectron spectroscopy (XPS) for Ti $2p_{3/2}$ and O 1s for TBT-HIO_3_50 before (**a**,**b**) and after phenol photodegradation (**c**,**d**).

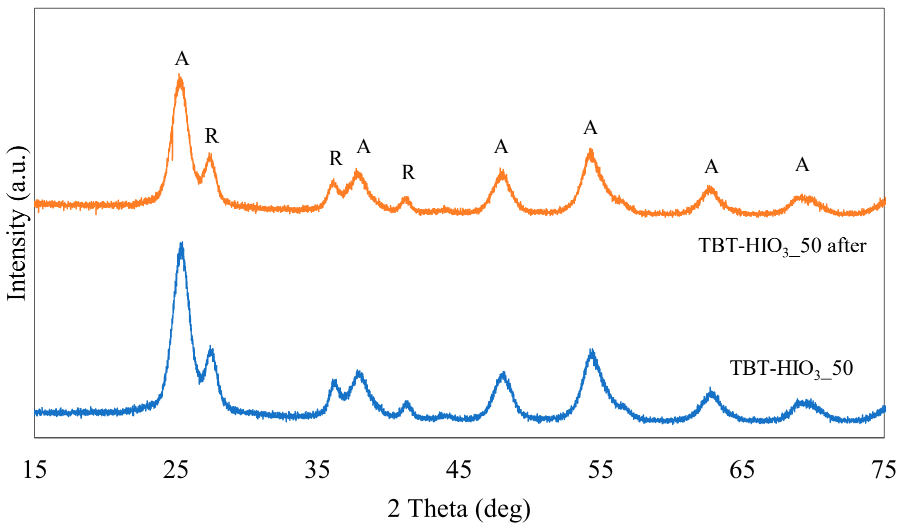

Figure S3. XRD patterns for defective TBT-HIO_3_50 photocatalyst before and after a photocatalytic phenol degradation (A–anatase, R–rutile).

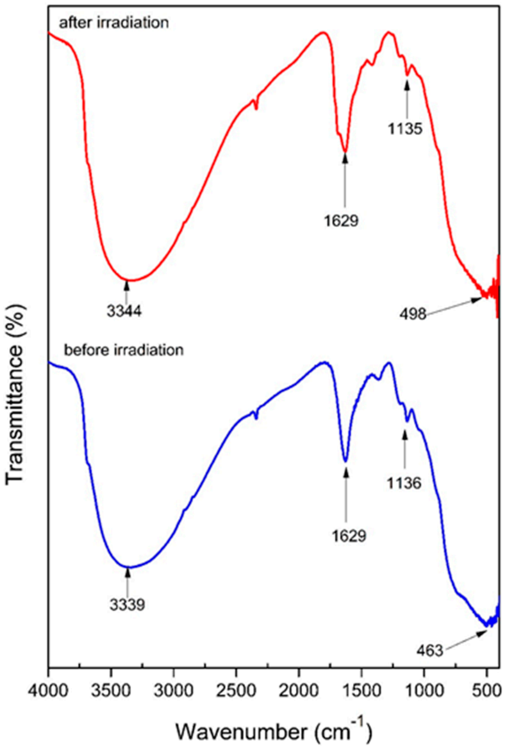

Figure S4. Fast-Fourier transformation spectroscopy (FTIR) spectra of defective TBT-HIO_3_50 before and after phenol photodegradation.

Table S1. Fraction of oxidation states of Ti as well as surface composition of defective TBT-HIO_3_50 photocatalyst before and after phenol degradation.

**Photocatalyst**	**Ti 2p$_{3/2}$ (%)**		**O 1s (%)**
	**Ti^{4+}**	**Ti^{3+}**	
TBT-HIO_3_50	26.94	1.23	71.83
TBT-HIO_3_50 after	26.74	1.45	71.81

© 2020 by the authors. Licensee MDPI, Basel, Switzerland. This article is an open access article distributed under the terms and conditions of the Creative Commons Attribution (CC BY) license (http://creativecommons.org/licenses/by/4.0/).
